# Semiochemical-baited traps as a new method supplementing light traps for faunistic and ecological studies of Macroheterocera (Lepidoptera)

**DOI:** 10.1038/s41598-024-71109-8

**Published:** 2024-08-30

**Authors:** Szabolcs Szanyi, Attila Molnár, Kálmán Szanyi, Miklós Tóth, Júlia Katalin Jósvai, Zoltán Varga, Antal Nagy

**Affiliations:** 1 Present Address: Institute of Plant Protection, Faculty of the Agricultural and Food Sciences and Environmental Management, University of Debrecen, P. O. Box 400, Debrecen, 4002 Hungary; 2 For the Nature- and Environmental Protection–PAPILIO (NGO), Molodizhna st. 41, Velyka Dobron’, 89463 Ukraine; 3 Department of Zoology and Ecology, Hungarian University of Agriculture and Life Sciences, Páter Károly str. 1, 2100 Gödöllő, Hungary; 4 Plant Protection Institute, HUN-REN CAR, P. O. Box. 102, Budapest, 1525 Hungary; 5 Department of Evolutionary Zoology and Human Biology, University of Debrecen, Egyetem tér 1, 4032 Debrecen, Hungary

**Keywords:** Agroecology, Biodiversity, Forest ecology, Chemical ecology, Entomology

## Abstract

Attractivity and selectivity of two types of traps with synthetic, long-lasting, bisexual generic attractants were compared to conventional light traps to promote their wider use, as an easy-to-use standardised method for entomology. The targeted herbivorous Macroheterocera species playing important role in ecosystems as food source for higher trophic levels (e.g. predatory arthropods, birds and mammals), while other hand they can cause significant economic loss in agriculture. Data on their population dynamic and composition of their assemblages are necessary for both nature conservation and efficient pest management. Light- and semiochemical-baited traps with semisynthetic- (SBL = the acronym stands for semisynthetic bisexual lure) and synthetic lures (FLO = the acronym stands for floral lure of synthetic floral compounds) were used in species rich area of West Ukraine, and in all 10,926 lepidopterans trapped were identified. The attractivity of the light trap was highest with 252 species caught, while traps with semiochemicals captured 132 species including 28 exclusively caught only by them. The qualitative selectivity of light vs. semiochemical-baited traps differed considering both taxa and habitat preferences in such a way that they completed each-other. Differences in quantitative selectivity were also proved even in case of pest species. The parameters of methods varied depending on the phenological phases of the studied assemblages. Considering the revealed attractivity and selectivity, the parallel use of the two methods can offer improved reliable data for conservation biology and pest management.

## Introduction

Various types of light- and semiochemical-baited traps have been used for the monitoring of night-active insects and for other entomological surveys for decades. The main target group of these experiments is the Macroheterocera (traditionally “larger moths” including e.g. Bombycoidea, Geometroidea, Noctuoidea, etc.) containing both high number of economically important pests (e.g. silk moths, hawk moths, loopers, cutworm moths, etc.) including invasive alien species, and rare and/or protected ones (e.g. *Dioszeghyana schmidtii, Arytrura musculus* listed in Natura 2000 and EU Habitat Directive Annex II and IV). Considering their diversity and abundance they play an important role in local food webs as herbivorous providing rich food source for parasitic and predatory groups and serve as sensitive indicators of environmental change. Survey and monitoring of their population is important for both nature conservation purposes and effective and sustainable plant protection. Regarding the limits and biases of widely used methods (e.g. light- and sex-pheromone traps) there is an urgent need for the development of comparable, reliable and profitable new methods.

The first effective light traps were used in Great Britain^1^ and North America^2^, while in Hungary a country-wide light trap network was established for plant protection purpose, and some years later, another one for forest pest monitoring^3,4^. Actual and long-term data sets provided by light traps have been used not only in plant protection but also in faunistic and ecological surveys e.g., for the evaluation of the recent climate change^4–13^.

However, different types of light traps are differing in selectivity and efficiency, depending both on wavelength and several environmental factors which limit the use of the collected data. Their efficiency highly depends on the phototactic activity of insects which is strongly influenced by the light colour, physiological factors of the given individuals (e.g., sexual activity, feeding) and several environmental factors such as temperature, air pressure, humidity, precipitation, disturbing light sources including moonlight, etc.^14–18^.

Numerous types of natural lures are also widely used in entomological studies. Different mixtures of natural ingredients including honey, fruit extracts and alcoholic drinks (beer, wine) have been used since the second half of the nineteenth century^19–22^. They have become popular, however, in the monitoring schemes they had been overshadowed by the modern, transportable light sources developed since the end of the last century^23–26^. Another disadvantage of such lures is that they are usually active only for some days or even less in field conditions.

After the discovery of the first pheromone (Bombykol) in 1959 sex pheromone traps have become widely used in insect monitoring. Although they often also capture some specimens of non-target species^27–29^, they can be considered as species-specific. Since only males are attracted by them, they are unsuitable for signalizing the sex-ratios and female swarming^30,31^.

Most recently, new types of synthetic lures have been developed using other semiochemicals. One of the first compounds studied is phenylacetaldehyde, which proved to be attractive for both sexes of many species of noctuid moths^32^. Later, several cutworm and armyworm pest species (e.g., *Xestia c-nigrum, Mamestra configurata* and *Lacanobia subjuncta*), and grass looper species (*Mocis* spp.) were also successfully captured with phenylacetaldehyde-baited traps^33^. In Alaska, efficiency of traps baited with multicomponent lures including phenylacetaldehyde, methyl salicilate, methyl-2-methoxybenzoate and β-myrcene were ascertained for Noctuidae species^34^.

The effectiveness of the combination of acetic acid and isoamyl alcohol (3-methyl-1-butanol) was described in North America first^35,36^, while its attractivity for noctuids was investigated also in Europe, between 2001 and 2009^37^. During the last decade, the addition of different synthetic compounds and natural ingredients (wine and beer) were intensively studied on the attractivity of both phenylacetaldehyde and isoamyl alcohol, and optimized lures (i.e. the SBL and FLO; for explanation of the acronyms refer to “Methods”, description of experimental lures) with a longevity of several weeks in field conditions were developed^37,38^.

During the development of the above bisexual (as opposed to pheromones, which only attract males in most cases) generic lures for noctuid pests, a wide range of moths living in semi-natural and natural habitats were caught as non-target species with these lures^39–41^. These species can be the targets of faunistic and ecological studies. The high number of species caught allows us to compare these data sets with a large set of light trap data, to evaluate the potential use of the generic semiochemical-based lures beyond their original purpose.

To carry out this comparison, in 2015, the SBL and FLO as two types of generic semiochemical-based lures and a light trap (of Jermy type) were used parallelly, in the margin of the Velyka Dobron’ forest (Ukraine, Transcarpathian region), of which the nocturnal Macroheterocera fauna was well-known due to a former, 5-year-long, intensive light trap sampling^40^.

During the investigation, the attractivity and selectivity of traps baited with the different lures, and a light trap were assessed and compared, regarding both qualitative- (species-) and quantitative composition of samples. Additionally, the effect of the phenology on attractivity was also evaluated to provide data for more appropriate use of the tested methods.

## Results

### Attractivity of different traps

**Fig. 1:**
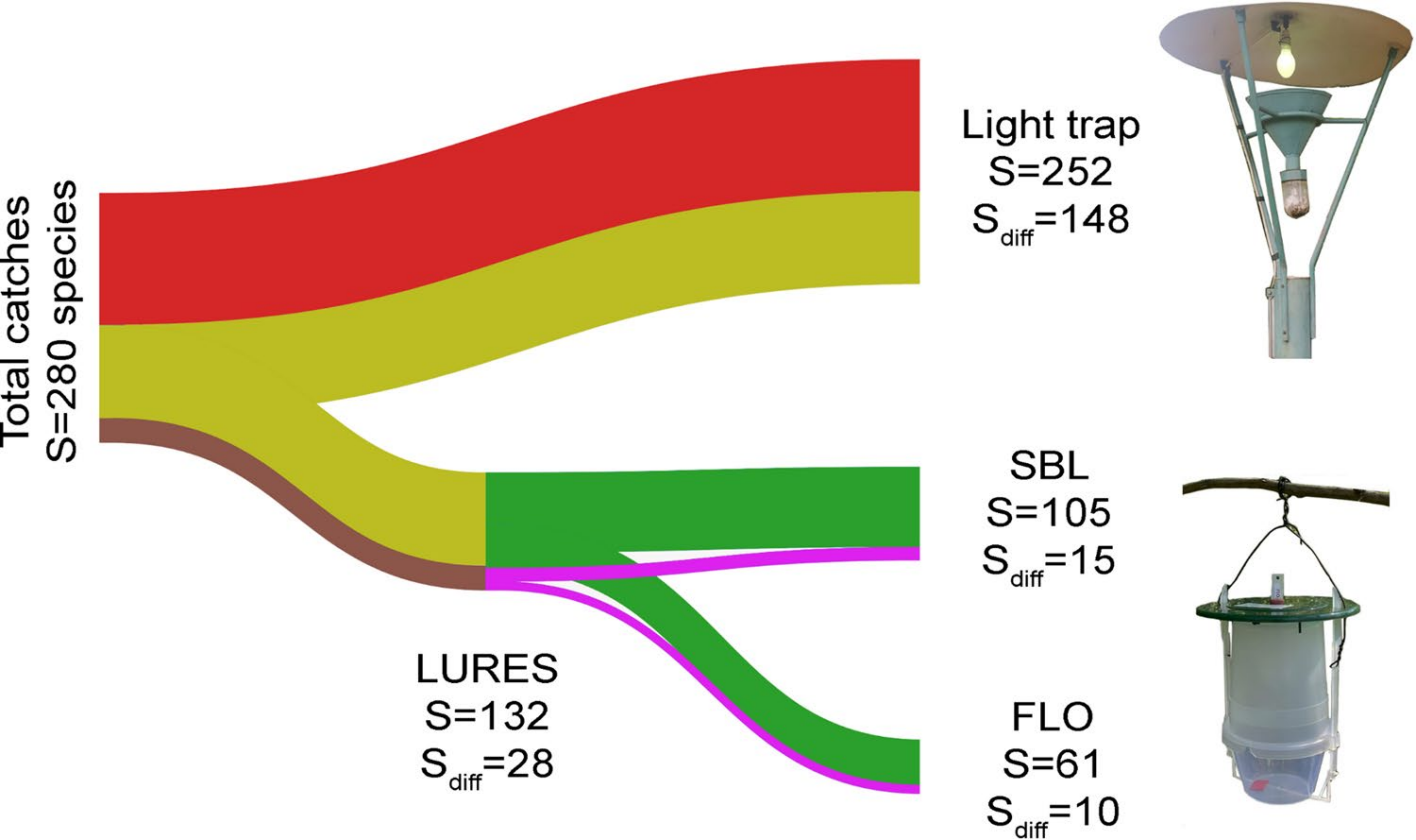
Distribution of species among different trap types (see also Tables 1 and 3). SBL: semisynthetic bisexual lure, FLO: phenylacetaldehyde-based lure, S: species richness, S_diff_: number of species caught exclusively by the given trap type.

**Fig. 2:**
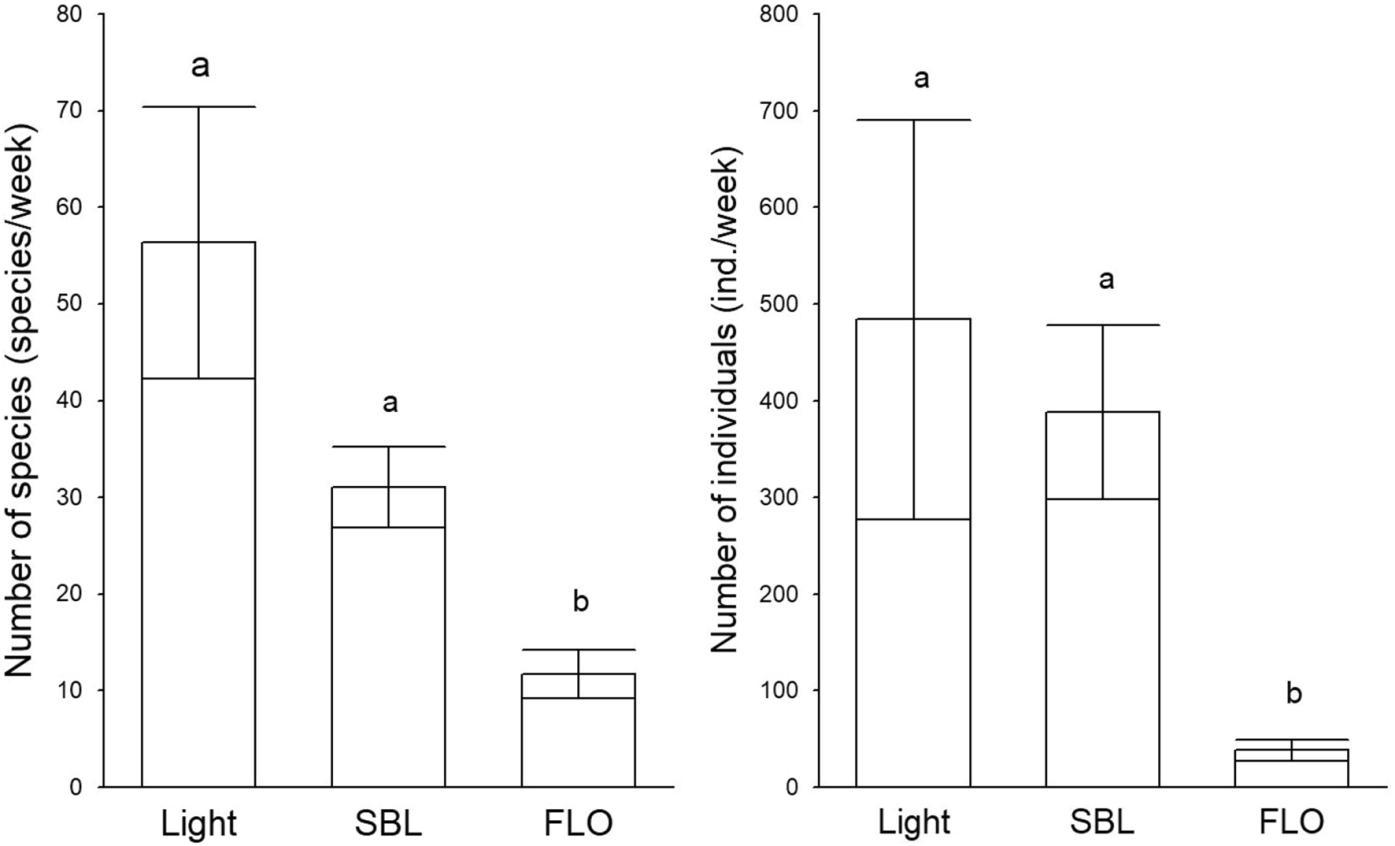
Mean number of caught species and individuals per week (± SE) collected with different tested trap types. Small letters refer to significant differences based on Mann–Whitney U test (P < 0.05).

During the samplings (02.08.2015–25.10.2015), 280 Macroheterocera species (families see in Table 1), containing formerly not reported species from the area, were caught. The total species-richness and number of species belonging to different families and subfamilies depended on the treatments (Table 1). The light trap showed the widest effect range with 252 species caught (90.0% of all observed species) of nine families. Lures attracted 132 species of six families. The effect range of the lures tested also differed: SBL (Semisynthetic Bisexual Lure) lure attracted 105 species of six families, while FLO (Floral) lures attracted 61 species of only three families (Table 1, Fig. 1) (for explanation of abbreviations SBL and FLO please refer to “Methods”). The mean species-richness was also highest in case of the light trap, but it did not differ significantly from the species-richness provided by the SBL lures. FLO lure attracted significantly less species than the other two treatments (Fig. 2). The traps with lures mainly caught species of Noctuidae and Erebidae families with ratios of 70.5% and 13.6%, respectively (Table 1).

**Table 1:** The total number of species caught (S_Total_) and number (S) and ratio (%) of species belonging to different Lepidoptera families and Erebidae and Noctuidae subfamilies by trap types and in the whole sample (SUM).

	Light trap		SBL		FLO		LURES		SUM	
	S	%	S	%	S	%	S	%	S	%
S_Total_	252		105		61		132		280	
Lasiocampidae	5	2.0	0	0.0	0	0.0	0	0].0	5	1.8
Sphingidae	8	3.2	1	1.0	0	0.0	1	0.8	8	2.9
Drepanidae	4	1.6	0	0.0	0	0.0	0	0.0	4	1.4
Thyatiridae	5	2.0	3	2.9	0	0.0	3	2.3	5	1.8
Geometridae	58	23.0	5	4.8	15	24.6	16	12.1	63	22.5
Notodontidae	14	5.6	0	0.0	0	0.0	0	0.0	14	5.0
Nolidae	8	3.2	1	1.0	0	0.0	1	0.8	8	2.9
Erebidae	31	12.3	14	13.3	12	19.7	18	13.6	38	13.6
Arctiinae	7	2.8	0	0.0	0	0.0	0	0.0	7	2.5
Lithosiinae	7	2.8	3	2.9	4	6.6	4	3.0	7	2.5
Catocalinae	2	0.8	6	5.7	1	1.6	7	5.3	7	2.5
Herminiinae	5	2.0	1	1.0	2	3.3	2	1.5	5	1.8
Lymantriinae	3	1.2	0	0.0	0	0.0	0	0.0	3	1.1
Others^a^	7	2.8	4	3.8	5	8.2	5	3.8	9	3.2
Noctuidae	119	47.2	81	77.1	34	55.7	93	70.5	135	48.2
Xyleninae	50	19.8	38	36.2	12	19.7	41	31.1	58	20.7
Hadeninae	19	7.5	13	12.4	3	4.9	14	10.6	20	7.1
Noctuinae	18	7.1	18	17.1	4	6.6	18	13.6	20	7.1
Plusiinae	6	2.4	2	1.9	9	14.8	9	6.8	9	3.2
Acronictinae	6	2.4	4	3.8	0	0.0	4	3.0	7	2.5
Heliothinae	4	1.6	2	1.9	2	3.3	2	1.5	4	1.4
Eustrotiinae	3	1.2	1	1.0	1	1.6	1	0.8	3	1.1
Acontiinae	3	1.2	0	0.0	0	0.0	0	0.0	3	1.1
Amphipyrinae	3	1.2	1	1.0	1	1.6	1	0.8	3	1.1
Others^b^	7	2.8	2	1.9	2	3.3	3	2.3	8	2.9

^a^Rivulinae, Boletobiinae, Aventiinae, Herminiinae, Hypeninae, Eublemminae, Phytometrinae, Scoliopteryginae.

^b^Pantheinae, Acronictinae, Metoponiinae, Cuculliinae, Amphipyrinae, Psaphidinae, Condicinae, Heliothinae, Bryophilinae, Eriopinae.

During the study, 10,857 Macroheterocera specimens were caught. The light trap captured more moths than traps with lures, but the difference was significant only in the comparison with FLO lures, and the SBL lure was also significantly more attractive than FLO lure (Fig. 2).

### Qualitative selectivity of methods

The light trap proved to be effective for attracting Arctiinae and Lymantriinae (Erebidae family) species, which were only caught with this treatment, while it was less attractive for Catocalinae species (caught less than 30% of the species collected by traps with lures). In the case of other Erebidae subfamilies, the efficiency of the lures was more than 50% lower than the efficiency of the light trap (Table 1).

Regarding the Noctuidae family, remarkable differences were found among subfamilies. In the case of Acronictinae*,* Heliothinae*,* Eustrotiinae and Amphipyrinae subfamilies, the light trap proved to be much more efficient than the treatments with lures. Considering the most diverse Xyleninae, Hadeninae and Noctuinae subfamilies, nearly or completely the same number of species were caught by light traps and traps with lures, mainly due to the wide range of attractivity of the SBL lure. Exceptionally, the FLO traps showed higher attractivity for Plusiinae species than both the light and SBL-baited traps (Table 1). PCA analysis based on relative frequencies of families, and subfamilies of the Noctuidae family showed similar attractivity of light- and SBL-baited traps for the species of Xyleninae and Noctuinae subfamilies and Erebidae family. Considering the abundance of the species, this pattern was caused by the high catches of *Trachea atriplicis*, *Allophyes oxyacanthae*, *Craniophora ligustri, Xestia c-nigrum* and *X. xanthographa*. Contrarily, FLO lure showed specificity for Plusinae subfamily, which was derived by high catches of *Autographa gamma* (Fig. 2).

Considering the habitat types, methods also showed different selectivity. In the whole fauna, species of deciduous forests were dominant (47.9%), followed by grassland species (27.9%) and generalists (21.1%). Ratio of migratory species was only 3.2% (Table 2).

**Table 2:** Number and ratio of Macroheterocera species belong to different ecotypes (faunal components; Varga et al.^78^) by trap types and in the whole sample (SUM).

	Light trap		SBL		FLO		LURES		SUM	
	S	%	S	%	S	%	S	%	S	%
Total number of species	252		105		61		132		280	
Generalist	53	21.0	32	30.5	18	29.5	40	30.3	59	21.1
Migratory	8	3.2	4	3.8	3	4.9	5	3.8	9	3.2
Deciduous forest fauna	119	47.2	52	49.5	22	36.1	59	44.7	134	47.9
Silvicolous (s.l.)	59	23.4	33	31.4	12	19.7	36	27.3	67	23.9
Oakwood	12	4.8	7	6.7	2	3.3	7	5.3	13	4.6
Willow-poplar	29	11.5	10	9.5	4	6.6	11	8.3	32	11.4
Birch-alder	4	1.6	0	0.0	1	1.6	1	0.8	5	1.8
Pine-sprouce	1	0.4	0	0.0	0	0.0	0	0.0	1	0.4
Forest edge	3	1.2	1	1.0	0	0.0	1	0.8	3	1.1
Nemoral	11	4.4	1	1.0	3	4.9	3	2.3	13	4.6
Grassland fauna	72	28.6	17	16.2	18	29.5	28	21.2	78	27.9
Altoherbosa	8	3.2	1	1.0	5	8.2	5	3.8	9	3.2
Moor-marsh	20	7.9	5	4.8	3	4.9	7	5.3	22	7.9
Arundiphilous	7	2.8	0	0.0	0	0.0	0	0.0	7	2.5
Mesophilous	17	6.7	5	4.8	6	9.8	7	5.3	18	6.4
Steppic	20	7.9	6	5.7	4	6.6	9	6.8	22	7.9

**Fig. 3:**
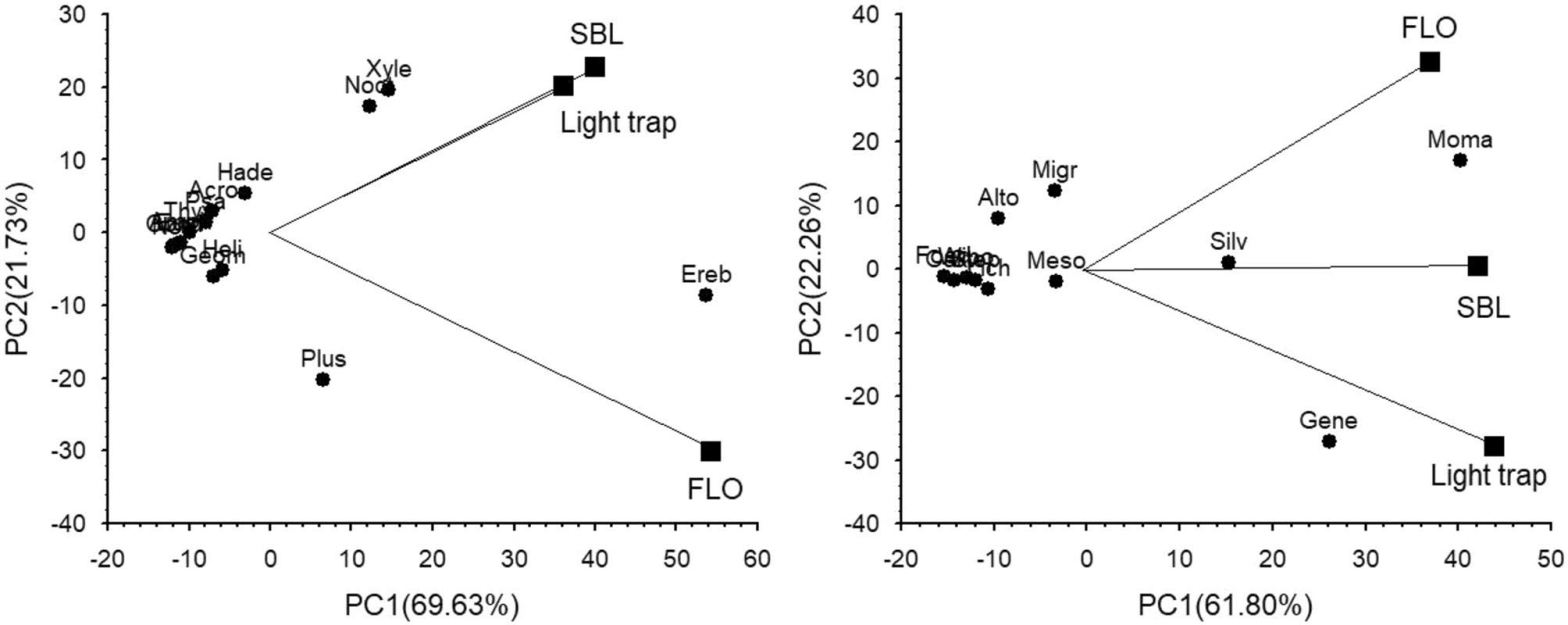
Biplots of principal component analysis (PCA) for tested trap types, Macroheterocera family and sub-family (Thya-Thyatiridae; Geom-Geometridae; Noli-Nolidae; Ereb-Erebidae; Noct-Noctuidae; Xyle-Xyleninae Hade-Hadeninae; Plus-Plusiinae; Acro-Acronictinae; Heli-Heliothinae; Eust-Eustrotiinae; Amph-Amphipyrinae) and eco-groups (Gene-Generalist; Migr-Migratory; Silv-Silvicolous; Oakw-Oakwood; Wipo-Willow-poplar; Bial-Birch-alder; Pisp-Pine-sprouce; Foed-Forest edge; Nemo-Nemoral; Alto-Altoherbosa; Moma-Moor-marsh; Arun-Arundiphilous; Meso-Mesophilous; Step-Steppic) of species.

In the light trap catches, a clear dominance of species inhabiting deciduous forests (silvicolous (s.l.), nemoral, oakwood, willow-poplar, birch-alder, forest edge) was observed (47.2%). The ratio of grassland species (28.6%) was higher than the average, while the ratio of generalists was roughly average (21.0%) (Table 2). Considering the lures, SBL mainly attracted forest species (49.5%) with slightly higher ratio than the light trap did. This high ratio was mainly derived by the high ratio of „common” deciduous forest (silvicolous) species (31.4%), followed by relatively high ratio of generalists (30.5%) and low ratio of grassland species (16.2%). Birch-alder specialists and species of coniferous forests (pine-spruce) were not or just sparsely captured by traps with lures. In FLO-baited traps, species of deciduous forests showed lower ratio (36.1%), while generalists and grassland species were relatively frequent (both were 29.5%). In this treatment, grassland species were mainly represented by species of altoherbosa and mesophilous eco-groups. Comparing with the ratios measured in the whole fauna, light trap did not show selectivity. In general, lures caught a higher ratio of generalist species, while the ratio of deciduous forest species was higher in SBL lures, and the migratory and grassland species preferred FLO lure (Table 2). Based on PCA analysis calculated with the relative frequencies of taxa and eco-groups, the light trap was more attractive for generalists, SBL lure for silvicolous and the two lures equally for moor-marsh eco-groups (Fig. 3).

The selectivity of treatments was characterized also by the number and ratio of differential species. Their number was 148 in the light trap, which was 58.7% of the total sample (Fig. 1, Table 3). Lasiocampidae and Drepanidae species were only caught with this treatment, but more than 80% of caught Sphingidae, Geometridae, Notodontidae and Nolidae species were also trapped exclusively with it (Table 3). Other Geometridae species proved to be specific for traps with lures and some of them were even attracted only to SBL (e.g., *Idaea dimidiata*) or FLO (e.g., *Xanthorhoe quadrifasciata*, *Ligdia adustata* and *Cabera pusaria*) lures. In the case of SBL lures, the ratio of differential species was lower (20.0%) than in FLO lures (26.7%). Considering the Erebidae family, the ratio of differential species was also highest in light traps (64.5%, 20 species). For lures together, 28 differential species were recorded, from which 7 (38.9%) belonged to the Erebidae. In SBL lures, more differential Erebidae species were found (*Lygephila pastinum*, *Catocala nupta*, *C. electa*, *C. sponsa*) than in FLO lures, which caught only one Erebidae species (*Euclidia glyphica*) exclusively (Table 3).

**Table 3:** Number of differential species (S_diff_) belonging to different families and Erebidae and Noctuidae subfamilies and their ratio (%) compared to the total number of species caught by trap types and volatile together.

	Light trap		SBL		FLO		LURES	
	Sdiff	%	Sdiff	%	Sdiff	%	Sdiff	%
S_diff_ total	148	58.7	15	14.3	10	16.4	28	21.2
Lasiocampidae	5	100.0	–	–	–	–	–	–
Sphingidae	7	87.5	0	0.0	–	–	0	0.0
Drepanidae	4	100.0	–	–	–	–	–	–
Thyatiridae	2	40.0	0	0.0	–	–	0	0.0
Geometridae	47	81.0	1	20.0	4	26.7	5	31.3
Notodontidae	13	92.9	–	–	–	–	–	–
Nolidae	7	87.5	0	0.0	–	–	0	0.0
Erebidae	20	64.5	4	28.6	1	8.3	7	38.9
Rivulinae	0	0.0	0	0.0	0	0.0	0	0.0
Boletobiinae	1	100.0	–	–	–	–	–	–
Aventiinae	2	100.0	–	–	–	–	–	–
Herminiinae	3	60.0	0	0.0	0	0.0	0	0.0
Hypeninae	0	0.0	0	0.0	0	0.0	1	50.0
Eublemminae	0	0.0	–	–	0	0.0	0	0.0
Phytometrinae	1	100.0	0	–	–	–	–	–
Scoliopteryginae	–	–	0	0.0	0	0.0	1	100.0
Lymantriinae	3	100.0	–	–	–	–	–	–
Arctiinae	7	100.0	–	–	–	–	–	–
Lithosiinae	3	42.9	0	0.0	0	0.0	0	0.0
Catocalinae	0	0.0	4	66.7	1	100.0	5	71.4
Noctuidae	42	35.3	10	12.3	5	14.7	16	17.2
Plusiinae	0	0.0	0	0.0	3	33.3	3	33.3
Eustrotiinae	2	66.7	0	0.0	0	0.0	0	0.0
Acontiinae	3	100.0	–	–	–	–	–	–
Pantheinae	1	100.0	–	–	–	–	–	–
Acronictinae	3	50.0	1	25.0	–	–	1	25.0
Metoponiinae	1	100.0	–	–	–	–	–	–
Cuculliinae	–	–	–	–	1	100.0	1	100.0
Amphipyrinae	2	66.7	0	0.0	0	0.0	0	0.0
Psaphidinae	0	0.0	0	0.0	0	0.0	0	0.0
Condicinae	1	50.0	0	0.0	–	–	0	0.0
Heliothinae	2	50.0	0	0.0	0	0.0	0	0.0
Bryophilinae	1	100.0	–	–	–	–	–	–
Eriopinae	1	100.0	–	–	–	–	–	–
Xyleninae	17	34.0	6	15.8	1	8.3	8	19.5
Hadeninae	6	31.6	1	7.7	0	0.0	1	7.1
Noctuinae	2	11.1	2	11.1	0	0.0	2	11.1

The number and ratio of differential species belonging to the Noctuidae were the highest in the light trap samples (20 species, 64.5%). Acontiinae, Pantheinae, Metoponiinae, Bryophilinae and Eriopinae species were caught only with this treatment. In the highly diverse subfamilies, the ratio of differential species was remarkably lower: Xyleninae (34.0%), Hadeninae (31.6%) and Noctuinae (11.1%). There were no Plusiinae species captured exclusively by the light trap. Contrarily, some Noctuidae species proved to be differential for SBL lure (10 species) or FLO (5 species) lure. The specificity of SBL was revealed for six species of Xyleninae (*Dypterygia scabriuscula*, *Oligia strigilis*, *Xylena exsoleta*, *Conistra rubiginea*, *Conistra erythrocephala*, *Agrochola humilis*), for two of Noctuinae (*Euxoa obelisca*, *Agrotis ipsilon*) and for one species of Hadeninae (*Mythimna pudorina*) and another of Acronictinae (*Acronicta auricoma*) subfamilies. The FLO lure proved to be specific for three species of Plusiinae (*Abrostola asclepiadis*, *A. tripartita*, *Trichoplusia ni*) and for one species of Cuculiinae (*Cucullia umbratica*) and Xyleninae (*Lithophane semibrunnea*) subfamilies each (Table 3).

Considering habitat types of the differential species, treatments also showed selectivity. Light trap and SBL lure were similarly selective for deciduous forest species, since the ratios of silvicolous and nemoral habitat type components were 50.7% and 60.0%, respectively. SBL lure showed low selectivity for grassland species, while in this point of view FLO showed nearly the same rate of selectivity (30.0%) as light trap (33.8%) did. FLO lure had equal selectivity for species of grasslands and deciduous forests (40.0%). In SBL lures, the ratio of silvicolous (40.0%) and oakwood (6.7%) habitat types were higher than in other trap types. The FLO lure showed relatively high specificity for differential species of birch-alder specialists and altoherbosa components (Table 4).

**Table 4:** Number of differential species (S_diff_) belonging to different ecotypes and their ratio (%) compared to the total number of species caught by trap types and volatile traps together (LURES).

	Light trap		SBL		FLO		LURES	
	Sdiff	%	Sdiff	%	Sdiff	%	Sdiff	%
Generalist	19	12.8	3	20.0	2	20.0	6	21.4
Migratory	4	2.7	0	0.0	1	10.0	1	3.6
**Decidous forest fauna**	**75**	**50.7**	**9**	**60.0**	**4**	**40.0**	**15**	**53.6**
Silvicolous (s.l.)	**31**	**20.9**	**6**	**40.0**	**2**	**20.0**	**8**	**28.6**
Oakwood	6	4.1	1	6.7	0	0.0	1	3.6
Willow-poplar	21	14.2	2	13.3	0	0.0	3	10.7
Birch-alder	4	2.7	0	0.0	1	10.0	1	3.6
Pine-sprouce	1	0.7	0	0.0	0	0.0	0	0.0
Forest edge	2	1.4	0	0.0	0	0.0	0	0.0
Nemoral	10	6.8	0	0.0	1	10.0	2	7.1
**Grassland fauna**	**50**	**33.8**	**3**	**20.0**	**3**	**30.0**	**6**	**21.4**
Altoherbosa	4	2.7	0	0.0	1	10.0	1	3.6
Moor-marsh	15	10.1	1	6.7	1	10.0	2	7.1
Arundiphilous	7	4.7	0	0.0	0	0.0	0	0.0
Mesophilous	11	7.4	1	6.7	0	0.0	1	3.6
Steppic	13	8.8	1	6.7	1	10.0	2	7.1

Significant values are in bold.

### Quantitative selectivity of methods

**Fig. 4:**
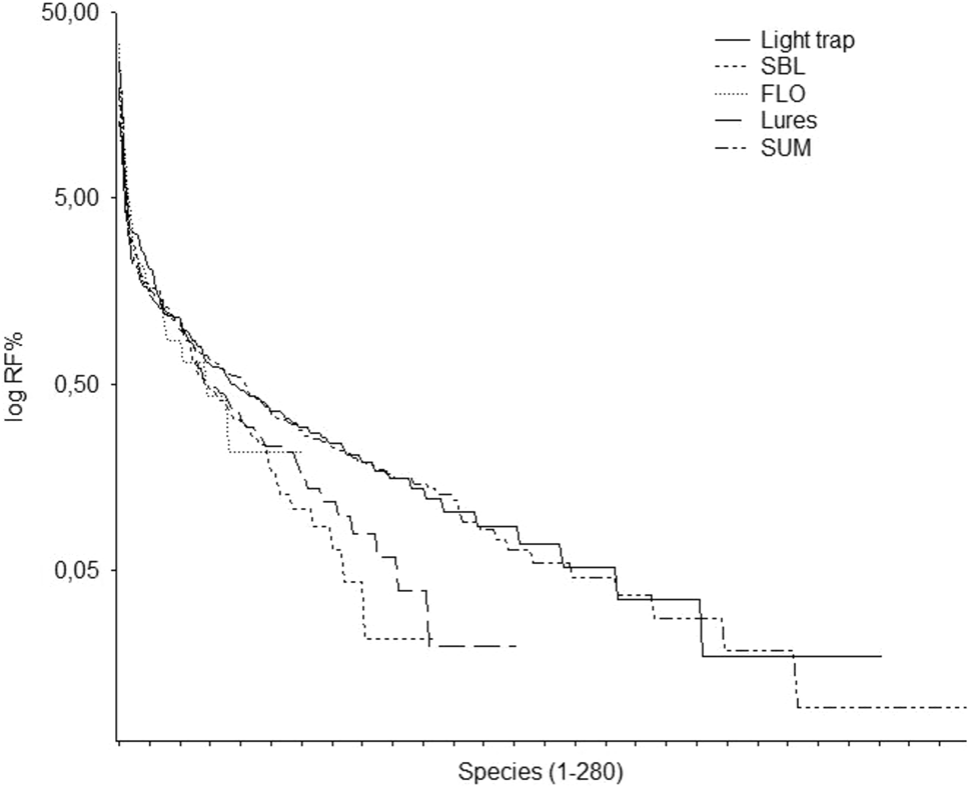
Dominance rank structure of Macroheterocera samples collected with different trap types (light trap and SBL and FLO lures), lures together and the whole sample.

The dominance rank structure of the samples taken with different methods also differed. Although the rank abundance curves showed lognormal distribution independently from the methods (Fig. 4), but the order of species was quite different. Kendall’s coefficient of concordance (W) showed high similarity of species ranks between samples of light-traps and SBL lures and also between light- and traps with SBL and FLO lures together, however only in case of the five most dominant species. SBL lures and FLO lures pooled together also provided similar rank structures. In other cases, the rank structure of samples taken with different methods showed low similarity (Table 5).

**Table 5:** Values of Kendall’s concordance (W) for the first 5, 10, 20, and all 44 species reached the 1% mean relative frequency in the whole sample.

	5			10			20			44		
	SBL	FLO	Lures	SBL	FLO	Lures	SBL	FLO	Lures	SBL	FLO	Lures
Light	0.936	0.577	0.950	0.727	0.406	0.676	0.545	0.283	0.465	0.559	0.334	0.478
SBL		0.553	0.987		0.381	0.980		0.516	0.977		0.417	0.959
FLO			0.577			0.415			0.609			0.556

Comparing with the mean rank of the species, light trap overestimated the rank of the dominant *Xestia c-nigrum*, and the subdominant *Craniophora ligustri*, *Lithosia quadra*, *Phragmatobia fuliginosa*, *Acontia trabealis* and *Athetis gluteosa*, which are eurytopic and widely distributed Palearctic species. Additionally, the rank of the locally rare (RF < 1%) *Ochropleura plecta*, *Axylia putris*, *Tholera cespitis*, *Nola aerugula*, *Lomaspilis marginata*, *Chiasmia chlatrata* and *Wittia sororcula* were also overestimated. Parallelly, the rank of 7 of the 17 most abundant species, including important pests (*Autographa gamma*, *Macdonnoughia confusa*), and further 16 locally rare species (e.g., *Cosmia affinis*, *Ectropis crepuscularia*, *Diachrysis chrysitis*, *Cirrhia icteritia*, *Amphipyra pyramidea* and *Conistra vaccinii* etc.) were highly underestimated. The SBL lures overestimated the ranks of three subdominant (*Allophyes oxyacanthae*, *Xestia xantographa* and *Hypena proboscidalis*) and nine locally rare species (e.g., *Cosmia affinis*, *Atethmia centrago*, *Cirrhia icteritia* etc.). The number of underestimated dominant and subdominant species was five while among the locally rare ones, six got lower rank in samples of SBL lures. The FLO lures overestimated the ranks of five abundant and 10 locally rare species including the economically important *Helicoverpa armigera*, and six species, *Autographa gamma*, *Macdonnoughia confusa, Abrostola triplasia*, *A. tripartita*, *Diacrysia chrysitis* and *D. stenochrysis* which belong to the Plusiinae subfamily. The number of underestimated species was six among the abundant species and two among the rare species consecutively (Table 6).

**Table 6:** The ranks and RF% of species with higher mean RF than 1% in the whole sample (light + SBL + FLO; 1–17) and species reached 1% RF in samples taken with different methods (light, SBL and FLO separately) alone and two lures together (Lures).

Species	Sum		Light		SBL		FLO		Lures	
	Rank	RF%	Rank	RF%	Rank	RF%	Rank	RF%	Rank	RF%
Mean RF > 1%										
* Pelosia muscerda*	1	24.26	1	12.92	1	26.04	1	33.84	1	26.74
* Trachea atriplicis*	2	6.37	52.5↓	0.36	2	18.54	49↓	0.22	2	16.88
* Xestia c-nigrum*	3	4.30	2↑	10.07	5	2.60	49↓	0.22	5	2.38
* Autographa gamma*	4	3.60	78↓	0.21	93.5↓	0.02	2↑	10.56	21.5↓	0.98
* Abrostola triplasia*	5	2.96	222.5↓	0.02	93.5↓	0.02	3↑	8.84	25↓	0.82
* Allophyes oxyacanthae*	6	2.48	104.5↓	0.12	3↑	6.90	33↓	0.43	3↑	6.31
* Xestia xanthographa*	7	2.30	14↓	1.48	4↑	4.77	25.5↓	0.65	4	4.40
* Craniophora ligustri*	8	2.10	3↑	4.46	9	1.85	–	–	9	1.68
* Helicoverpa armigera*	9	2.02	13	1.65	61↓	0.11	5↑	4.31	31.5↓	0.49
* Macdunnoughia confusa*	10	1.79	78↓	0.21	–	–	4↑	5.17	33↓	0.47
* Lithosia quadra*	11	1.73	5↑	3.41	23↓	0.92	19	0.86	23↓	0.92
* Hypena proboscidalis*	12	1.54	43↓	0.45	7↑	2.02	8.5↑	2.16	7↑	2.03
* Mythimna albipuncta*	13	1.31	12	2.03	18	1.25	25.5↓	0.65	16	1.19
* Phragmatobia fuliginosa*	14	1.20	4↑	3.60	–	–	–	–	–	–
* Noctua janthina*	15	1.14	19.5	1.14	13	1.63	25.5↓	0.65	12	1.54
* Acontia trabealis*	16	1.09	6↑	3.27	–	–	–	–	–	–
* Athetis gluteosa*	17	1.06	7↑	3.12	72.5↓	0.06	–	–	89↓	0.06
RF% > 1 at leas with one method										
* Cosmia affinis*	18	0.95	65.5↓	0.28	11↑	1.72	19	0.86	10↑	1.64
* Noctua pronuba*	19	0.92	37↓	0.53	19	1.16	16	1.08	19	1.15
* Ochropleura plecta*	20	0.90	8↑	2.60	61↓	0.11	–	–	75↓	0.10
* Ectropis crepuscularia*	21	0.89	125.5↓	0.09	–	–	6↑	2.59	52.5↓	0.23
* Axylia putris*	22	0.86	9↑	2.55	93.5↓	0.02	–	–	117.5↓	0.02
* Atethmia centrago*	23	0.83	93↓	0.15	6↑	2.34	–	–	6↑	2.13
* Lacanobia oleracea*	24	0.83	25.5	0.86	13↑	1.63	–	–	14	1.48
* Diachrysia chrysitis*	25	0.82	125.5↓	0.09	–	–	7↑	2.37	57.5↓	0.21
* Cirrhia icteritia*	26	0.80	139.5↓	0.07	8↑	1.89	33	0.43	8↑	1.76
* Tholera cespitis*	27	0.79	11↑	2.07	42.5↓	0.30	–	–	46.5↓	0.27
* Eilema griseola*	28	0.75	113↓	0.10	–	–	8.5↑	2.16	59↓	0.20
* Nola aerugula*	29	0.75	10↑	2.24	–	–	–	–	–	–
* Hypomecis roboraria*	31	0.72	52.5↓	0.36	67.5↓	0.09	10↑	1.72	52.5↓	0.23
* Hypomecis punctinalis*	32	0.64	61.5↓	0.29	61↓	0.11	12.5↑	1.51	52.5↓	0.23
* Amphipyra pyramidea*	33	0.62	222.5↓	0.02	13↑	1.63	49↓	0.22	13↑	1.50
* Habrosyne pyrithoides*	34	0.61	36	0.55	16.5↑	1.29	–	–	17.5↑	1.17
* Thyatira batis*	35	0.60	155.5↓	0.05	10↑	1.74	–	–	11↑	1.58
* Mesapamea secalis*	37	0.55	82.5↓	0.19	15↑	1.46	–	–	15↑	1.33
* Diachrysia stenochrysis*	38	0.55	99↓	0.14	–	–	12.5↑	1.51	65↓	0.14
* Lomaspilis marginata*	39	0.55	15↑	1.43	–	–	49↓	0.22	117.5↓	0.02
* Camptogramma bilineata*	40	0.54	113↓	0.10	93.5↓	0.02	12.5↑	1.51	61.5↓	0.16
* Epione repandaria*	41	0.51	178.5↓	0.03	–	–	12.5↑	1.51	65↓	0.14
* Chiasmia chlathrata*	42	0.49	16↑	1.26	–	–	49	0.22	117.5↓	0.02
* Conistra vaccinii*	46	0.44	222.5↓	0.02	16.5↑	1.29	–	–	17.5↑	1.17
* Abrostola tripartita*	47	0.43	–	–	–	–	15↑	1.29	70↓	0.12
* Wittia sororcula*	48	0.40	17↑	1.21	–	–	–	–	–	–
* Timandra comae*	55	0.36	78↓	0.21	–	–	19↑	0.86	81.5↓	0.08

↓: underestimated rank comparing the mean rank, ↑: overestimated rank comparing the mean rank.

Considering the pooled catches of the two lures tested, the ranks of two abundant (*Allophyes oxyacanthae* and *Hypena proboscidalis*) and eight locally rare species (e.g., *Cosmia affinis*, *Atethnia centrago*, *Cirrhia icteritia*, etc.) were overestimated. Contrarily, the ranks of six abundant (e.g., *Athetis gluteosa*, *Lithosia quadra*, *Abrostola triplasia*, etc.) and 15 locally rare species were underestimated (Table 6).

### Temporal changes of catches and species assemblages

Temporal changes in the number of species and individuals caught were remarkable during the period studied. Both values decreased continuously and markedly till 13rd of September. At the same time the decrease of the Noctuidae species-richness was lower, but their abundance also showed a high decrease. After that, species-richness and abundance of noctuids increased, with a second peak in 20th of September, and then both parameters showed a slow decrease.

**Fig. 5:**
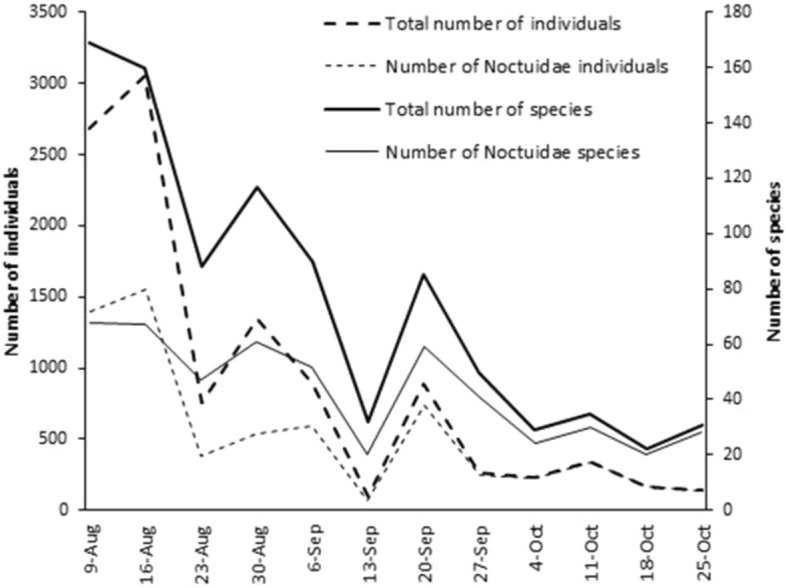
Temporal changes of the number of species and individuals in the whole sample and in case of Noctuids separately during the study period from 9th August to 25th October 2015.

Since not only the above parameters changed on 13th September, but the species composition as well, two phenological phases could be divided: summer up to 13th of September (6 samplings) and autumn started after that (to 25th of October, 6 samplings) (Fig. 5).

**Fig. 6:**
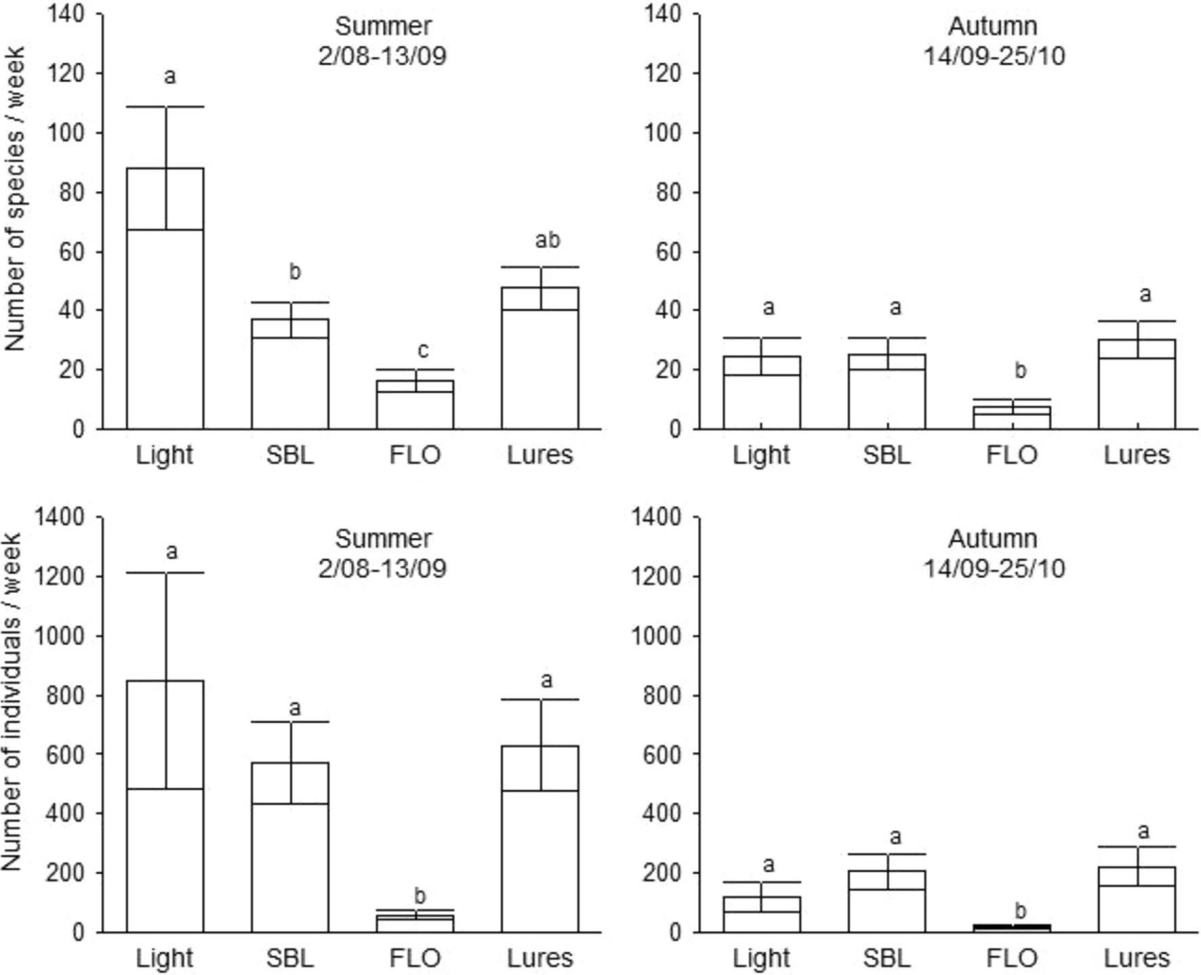
The mean number of caught species and individuals (± SE) by trap types and lures together in the summer (from 2/08 to 13/09) and autumn periods (from 14/09 to 25/10) in 2015. The small letters show significant differences between the trap types based on Mann–Whitney U-test, (P < 0.05).

In the two phenological phases, trap types showed different attractivity. In summer, the light trap collected significantly more species than traps with lures, and SBL lures were significantly more efficient than FLO lures. The two lures pooled together also attracted less species than the light trap alone, but the difference was not significant. In autumn, these differences mainly disappeared since the light- and SBL lures and the two lure types pooled together sampled nearly the same species number, however, FLO lure stayed significantly less attractive (Fig. 6).

The mean number of individuals caught showed a similar pattern as species-richness. In the summer, the light trap attracted more individuals than lures pooled together or kept separate, but the difference was significant only in case of the FLO lures, which caught the fewest individuals. Considering the abundances in autumn, the SBL lure was the most attractive and the FLO lure attracted significantly less specimens than the others (Fig. 6).

## Discussion

Light traps are the most widely used tools for faunistic and ecological entomological surveys and even for plant protection and forest entomological studies targeting night-active species independently from the habitat types (from agricultural to natural sites) and goals of the study (ecological, faunistic, population etc.)^42–44^. Since the efficiency and use of light traps is limited by environmental factors (e.g., temperature, lunar phase, light pollution, etc.) and features of the given trap type (wavelength, intensity, construction, etc.^18,26,41,45–47^), it has been combined with such additional methods as trapping with scent lures for a long time. These traditional scent lures complete the catches of light traps, but they are made from natural ingredients, based on unique, sometimes “secret” recipes of entomologists, and their efficiency and specificity were not documented^21,48^. Also, in most cases their efficiency changes within some days due to decomposition of the natural ingredients. In the pest monitoring, and pest control without pesticides (“mass trapping”, “lure and kill” and other related methods), the use of newly developed, standardised lures have become more important and common^38–40,49–52^. The present study attempts to establish their selectivity and efficiency of traps with two different synthetic, long-lasting (retained attractive activity for at least 4 weeks in field conditions), bisexual generic attractants, the SBL and FLO lures, and to compare them with those of light traps to suggest their application benefits in different entomological studies.

The efficiency formerly proved of traps with SBL or FLO lures was confirmed again. Attractivity of both baits was high considering both species number and abundances of Macroheterocera species, as in the case of our several former studies^39,39,53,54^.

Catches of traps with lures complemented the Macroheterocera check list provided by the widely used mercury-vapour light trap since the qualitative selectivity of the lures differed from each other and also from selectivity of the light-trap. Although species-richness measured with light trap (252 species) was higher than with different lures separately (SBL: 105 species, FLO: 61 species) and pooled together (132 species), considering mean catches of the samples, only the FLO traps showed lower efficiency than the other two methods. Additionally, lures could attract significant number of differential species (28 species: 15 for SBL and 10 for FLO), which were not caught by the light trap. Despite a former 5-year light trap sampling in the same area, the 1-year use of volatiles in 2014 could provide new data of 30 Noctuidae species, that shows the same trend in qualitative composition of the samples taken with different methods in the present study^40^. Although considering the widely used light traps there are not any analysis on the complementarity of the tested and even other semiochemical baited traps, our result showed that the SBL and FLO lures can significantly complete the Macroheterocera check list made with light traps.

On the other hand, the qualitative selectivity of the tested methods was also remarkably different at both the level of taxon and habitat preferences of species, that had never been analysed in the case of any lures before. Although the attractivity of the tested lures alone and in combination were tested in the case of many pest species^38,41,52,55–57^, the composition of the assemblages sampled have never been described. Light trap was selective for Notodontidae, Lasiocampidae and Drepanidae families and Arctiinae and Lymantriinae (Erebidae) subfamilies. Adults belonging to these subfamilies are aphagous^58^, which may explain that they were not attracted by feeding attractants. Contrarily, some Lithosiinae species which are daylight-active and regularly visit some nectar sources, e.g., *Sambucus ebulus* L. (and pers. obs.) appeared in the tested traps with lures with moderate species-richness and high abundance, thus this phenomenon needs further studies. Considering Noctuidae, more species of Acronictinae, Heliothinae, Eustrotiinae and Amphipyrinae were attracted to the light- than to the traps with lures. Pest species of Heliothinae, as *Helicoverpa armigera*, *Heliothis maritima*, are traditionally monitored with light traps^59–61^ which shows the efficiency of this method against them. In case of species of the more diverse Xyleninae, Hadeninae and Noctuinae subfamilies, the attractivity of lures together was nearly the same as that of light traps at species level. Comparing the two studied lures, SBL was selective for species belonging to Xyleninae, Hadeninae and Noctuinae subfamilies while FLO showed selectivity for species of Plusiinae subfamily. This pattern in this study confirmed the formerly described selectivity of these different baits^39,40,53,54^.

Considering the habitat preferences of moths, the light trap showed an intermediate character between the traps with lures. The SBL lures were more selective to the species of forested habitats, catching high ratio of silvicolous and oakwood species, while FLO lures were selective for birch-alder specialists and altoherbosa Noctuids. These differences of the lures tested have already been observed in different regions and habitat types, as well^40,53,54,62^. Based on the different selectivity of the two lures tested, the combined use of them can serve reliable data on the ecotype composition of the assemblages. In our case, the high ratio of deciduous forest fauna refers to the habitat structure of the sampling site which is one of the remaining patches of the former, extended forests of the Bereg Lowland, mixed with some humid open habitats, such as marshes and peatlands^63,64^.

The quantitative selectivity of the sampling methods (light vs. traps with lures) and lures tested were also revealed. Although the dominance-rank curves provided by different treatments were similar, the quantitative composition of the samples differed, even regarding some dominant and subdominant species. The traditional (Jermy type) light trap operated with mercury-vapour lamp overestimated the frequencies of both many widely distributed eurytopic Palearctic species (e.g., *Xestia c*-*nigrum*, *Craniophora ligustri*, *Lithosia quadra*, *Phragmatobia fuliginosa.* etc.), and some locally rare species, as *Ochropleura plecta*, *Axylia putris*, *Tholera cespitis*, *Nola aerugula*, etc. Contrarily, relative frequencies of nearly the half of the most abundant species (7/10) including economically important pests, such as *Autographa gamma* and *Macdunnoughia confusa*, were underestimated by this trap type. Considering the most dominant species, the SBL lures mainly underestimate the frequencies or provided values like average. FLO overestimated the relative frequencies of five abundant species including important pests: *Helicoverpa armigera*, *Autographa gamma* and *Macdunnoughia confusa*.

The sampling bias may lead to false decisions in conservation biology, forestry, and plant protection, where both the presence and abundance of species serve for the basis for decision making^65^. During monitoring surveys, one of the focal issues, how the habitat types and life history attributes are connected with the conservation, and also with the pest status of moth populations^66^. To draw right conclusions and make right decisions, the revealed selectivity and efficiency of the methods tested should be considered. In research and monitoring, different methods (various light traps, non-standardised baits, and their combinations) are used to characterise and compare Macroheterocera assemblages^67–69^. Although any kind of standardised methods can be suitable, the revealed differences in the selectivity of the methods tested showed that they describe the real quantitative composition with remarkable bias.

The traps with lures tested in this study could provide reliable data on swarming, even with only two checks of traps per week. Using the two bait types tested simultaneously, they could follow the temporal changes of the whole assemblage and the population dynamics of the most important pest species. In our study, the two characteristic phenological phases of the assemblages could be distinguished with them. Because of the standard composition of the traps, they can provide comparable data for both large temporal and spatial scales. Although the Jermy-type light traps are officially used in forest- and plant protection entomology since they are especially suitable for following population dynamics of pest species, their use is especially labour intensive, due to the high amount of sampled insects^3,70,71^.

Advantages, weaknesses and limits of different methods were assessed to promote the wider use of traps with SBL and FLO lures, as a new, easy to use and standardised method of ecological surveys on a wide range of Macroheterocera taxa. Our results confirm that the combined use of traditional light trap and different types of lure baits provide not only additional, new faunistic data, but also can reveal the real structural and functional composition of the moth (mostly Noctuidae) assemblages, thus, can strengthen the ecological background of biodiversity monitoring, conservation practices, forest entomology and plant protection forecast. Additionally, the traps with SBL or FLO lures used alone can be seen as an easy to use, less labour intensive, standardised alternative of light traps in both biological monitoring and pest management.

## Methods

### Study area

The samplings were carried out in the surroundings of Velyka Dobron’ (GPS: N48.4338°, E22.4041°), on the margin of the Velyka Dobron’ Forest and the former Szernye Marsh drained at the end of the nineteenth century. Recently, the area is mostly covered with a mosaic of secondary habitats and isolated patches of the original wetlands and forests. The Velyka Dobron’ Forest is an extended patch of an oak-ash-elm hardwood gallery forest, which is the most valuable natural habitat type of the region. The natural and semi-natural habitats preserve species-rich remains of the former, unique, and highly diverse wetland fauna until recent times^72^. On the other hand, at more xeric sites xerophilous silver lime (*Tilia tomentosa* Mill.)—oak forests, bushy forest fringes, forest clearings and willow scrubs can be found. The high habitat diversity of the area sustains highly abundant and species-rich Macroheterocera assemblages suitable for testing their most common sampling methods^62^.

### Trapping methods

The Jermy type fixed light trap operating with a 125 W mercury-vapour lamp located on the margin of a grassland and Velyka Dobron’ forest was the basis of the comparison. This trap type and its variants are generally used in faunistic and ecological studies, and even in plant protection and forest pest forecast and monitoring throughout the world^4^. According to the general methodology the light trap was used in every 2 days between 2nd of August and 25th of October in 2015. Samples taken in a given week were assumed to compare them with the samples of traps with lures taken in the same period.

CSALOMON^®^ VARL + funnel traps (Plant Protection Institute, HUN-REN CAR, Budapest, Hungary) containing semiochemical lures were used parallelly, at 300 m distance from the light trap to provide independency.

Four funnel traps were baited with SBL lure (a lure described in detail earlier^41,62,73^; the acronym SBL stands for “semisynthetic bisexual lure”—as opposed to pheromone lures which attract only one sex) containing isoamyl alcohol, acetic acid and red wine (1:1:1), evaporated from polypropylene tubes.

In another four funnel traps, the synthetic FLO lure was used, which contained phenylacetaldehyde, (*E*)-anethol, benzyl acetate and eugenol (1:1:1:1)^40,41^. (The acronym FLO stands for “floral lure”, since it contains synthetic floral compounds as active ingredients).

For unbaited controls, four funnel traps without any lure were also operated.

Experimental lures were custom-made for the purpose of the experiments, in the laboratory of Plant Protection Institute, HUN-REN CAR (Budapest, Hungary), as published earlier^41,62,73^.

Namely, for SBL lure, a custom-made polypropylene vial with lid (4 ml capacity, wall thickness 1 mm) was used. A dental roll (Celluron^®^, Paul Hartmann AG, Heidenheim, Germany) was placed into the vial, and 3 ml of the active ingredients (isoamyl alcohol, acetic acid and red wine; 1:1:1) was pipetted onto the dental roll. The lid of the vial was closed. When setting out to the field, a 4 mm hole was opened at the bottom of the vial, so that the compounds could evaporate into surrounding air. Isoamyl alcohol and acetic acid was obtained from Sigma-Aldrich Kft (Budapest, Hungary) and were > 95% pure as stated by the supplier. Red vine came from the vinery of Dr. Géza Vörös (Szekszárd, Hungary), deriving from joint preparation of Blaufrankisch (70%), Merlot (15%), Kadarka (7,5%) and Blauburger (7,5%) grapevines. Alcohol content: 13.6–13.8%, acid (acetic acid) content 0.4–0.6 g/l.

For the FLO lure polyethylene bag dispensers were used, their preparation was published earlier^37,41,62,73,74^. For preparing the dispensers a 1 cm piece of dental roll (Celluron^®^, Paul Hartmann AG, Heidenheim, Germany) was placed into a tight polyethylene bag made of 0.02 mm linear polyethylene foil. The dimensions of the polyethylene sachets were ca. 1.5 × 1.5 cm. The dispenser was attached to a plastic strip (8 × 1 cm) for easy handling when assembling the traps. For making up the baits, 0.4 ml of the blend of active ingredients of phenylacetaldehyde, (*E*)-anethol, benzyl acetate and eugenol (1:1:1:1)^40,41^ were administered onto the dental roll and the opening of the polyethylene bag was heat-sealed. When setting out to the field, active ingredients could evaporate through the PE walls of the dispenser. Active ingredents were obtained from Sigma-Aldrich Kft (Budapest, Hungary) and were > 95% pure as stated by the supplier.

Previous experience obtained in several years showed that in the field catches in both the SBL and the FLO-baited traps started to decrease after 4–5 weeks of field exposure. Therefore, in the present studies lures were replaced by new ones at 4-week intervals.

The moths caught were killed by Vaportape^®^ II insecticide strips developed especially for trapping insects (10% 2,2 dichlorovinyl dimethyl phosphate). Insecticide kills insects quickly and does not affect the attractivity of the baits. Each baited trap type was exposed in four repetitions (4 × 3 = 12 traps in total). These traps were hung on tree branches, at 20 m distance from each other, at the height of 1.8–2 m. They were checked and emptied once a week and were rotated weekly to mitigate the local effects on the catches.

The insect material collected was stored deep-frozen (at − 20 ºC) until identification at species level. The number of individuals caught by species were provided and the relative frequencies of species were also calculated.

The Noctuoidea taxa were identified according to Varga^75^. The taxonomic list follows the system of Lafontaine and Schmidt^76^, with the modifications of Zahiri et al.^77^. Regarding faunal elements and faunal components (habitat types), Varga et al.^78^ was followed.

### Data analysis

In order to evaluate the attractivity of the tested methods, measures of effect range and selectivity were used in the statistical analysis. The effect range of different traps was characterized by the total number of species caught, and number of species belonging to different families, and in the case of larger families, even to subfamilies. The selectivity was characterized by quantitative species composition and ratio of species at family and subfamily levels, and in the whole sample. The number of species and individuals caught was assessed on a weekly basis.

The number and ratio of differential species caught only by a given type of trap were also provided. The number and ratio of species and differential species belonging to different ecotypes were also calculated for each sampling method, for lures together and for the whole sample.

To characterize the selectivity of different sampling methods, the connections between the tested trap types and caught Lepidoptera families and ecotypes were analysed with principal component analysis (PCA).

In order to evaluate the effect of phenology on the attractivity of the tested trap types, temporal changes in the number of species and individuals were used regarding both the whole sample and the most abundant Noctuidae family. Based on these variables, a summer (before 13/09/2015) and an autumn (after 13/09/2015) period were separated. The mean species-richness and number of individuals of the samples collected in these periods were compared with Mann–Whitney U-test, since data did not fulfil the terms of the parametric test. The homogeneity of variances was tested with Levene-test, while normal distribution was checked with Q-Q plots.

The rank structure of the whole sampled material (based on mean RF%) and samples taken with different methods and lures together were visualized on log graph of RF%. The similarity of rank structures was analysed with Kendall’s Coefficient of Concordance (W), a non-parametric test calculating with abundance ranks of species^79^. Similar composition of samples taken with different methods results high and significant W value, while great variation of rank structure leads to lower W values. For calculations Statistica 7.0 Single User Version (http://www.statsoft.com) software was used.

## Data Availability

The data that support the findings of this study are openly available in Zenodo at 10.5281/zenodo.8126602, reference number https://zenodo.org/record/8126602.
